# Synthesis and Biological Activity of Silyl- and Germylsubstituted Trifluroacetylfurans

**DOI:** 10.1155/MBD.2001.211

**Published:** 2001

**Authors:** Luba Ignatovich, Dzintra Zarina, Irina Shestakova, Skaidrite Germane, Edmunds Lukevics

**Affiliations:** Latvian Institute of Organic Synthesis Aizkraukles 21 Riga LV-1006 Latvia

## Abstract

A series of silyl, germyl and alkyl substituted trifluoroacetylfurans has been synthesized under Friedel-Crafts electrophilic acylation conditions. Biological investigations have demonstrated that germyl derivatives of trifluoroacetylfuran are more toxic than the silicon analogues. 5-Triethylgermyl-2-trifluoroacetylfuran was the most toxic compound (LD_50_, 11.2 mg kg^-1^, *i.p.* for white mice), 200 times more toxic than the silicon analogue. 5-*t*-Butyl- and 5-trimethylsilyl-2-trifluroacetylfuran prolong the duration of ethanol anaesthesia by 220 and 140%. 5-Triethylgermyl-2-trifluroacetylfuran exibited high anesthetic activity in hexobarbital test (prolonged the duration by 137%). Some of compounds influenced muscle tone and locomotor coordination parameters. 5-Triethylgermyl-2-trifluomacetylfuran exibited analgesic activity (ED_50_, 0.9 mg kg^-1^).


### SYNTHESIS AND BIOLOGICAL ACTIVITY OF SILYL- AND GERMYLSUBSTITUTED TRIFLUROACETYLFURANS

Luba Ignatovich*, Dzintra Zarina, Irina Shestakova, Skaidrite Germane and Edmunds Lukevics

Latvian Institute of Organic Synthesis, Aizkraukles 21, Riga, LV-1006, Latvia <ign@osi.lv>

### ABSTRACT

A series of silyl, germyl and alkyl substituted trifluoroacetylfurans has been synthesized under Friedel-Crafts electrophilic acylation conditions. Biological investigations have demonstrated that germyl derivatives of trifluoroacetylfuran are more toxic than the silicon analogues. 5-Triethylgermyl-2-trifluoroacetylfuran was the most toxic compound ($LD_{50}$ 11.2 mg kg $^{-1}$, *i.p.* for white mice), 200 times more toxic than the silicon analogue. 5-*t*-Butyl- and 5-trimethylsilyl-2-trifluoroacetylfuran prolong the duration of ethanol anaesthesia by 220 and 140%. 5-Triethylgermyl-2-trifluroacetylfuran exibited high anesthetic activity in hexobarbital test (prolonged the duration by 137%). Some of compounds influenced muscle tone and locomotor coordination parameters. 5-Triethylgermyl-2-trifluoroacetylfuran exibited analgesic activity ($ED_{50}$ 0.9 mg kg $^{-1}$ ).

### INTRODUCTION

The introduction of the trifluoroacetyl moiety into aromatics and heteroaromatics has been generally realized by Friedel-Crafts acylation and by reaction of organomagnesium or organolithium reagents with ethyl trifluoroacetate, trifluoroacetic acid or its salts [1-7 and ref. therein].

Friedel-Crafts electrophilic acylation of aryl(hetaryl)silanes, -germanes and -stannanes was accompanied by *ipso*-substitution of trialkylsilyl, -germyl or -stannyl group. Reactions of this kind have gained currency in organic synthesis primarily due to the fact that they provide a unique possibility for regiospecific insertion of various groups (halogen, nitro, alkyl, acyl, etc.) into the aromatic or heterocyclic ring [8,9].

We have realized the electrophilic acylation of organometallic furans leading to corresponding 5-trialkylgermyl(silyl)-2-trifluoroacetyl derivatives.

On the other hand, trifluoromethyl ketones have generated much interest as components of potent enzyme inhibitors [10]. A series of trimethylsilylated aliphatic and aromatic trifluoromethylketones was synthesized and evaluated for anti-acetylcholinesterase activity. One compound in this series – 3-(trimethylsilyl)trifluoroacetylbenzene (MDL-73745, Zifrosilone) was selected for further investigation for the treatment of Alzheimer's disease [10-12].

The present investigation elucidates the role of the substituent, its influence on psychotropic and antitumor activity of 5-$R_3M$(M=C,Si,Ge)-substituted 2-trifluoroacetylfurans.

### MATERIALS AND METHODS

### Chemistry

$^1H$ NMR spectra were recorded on a Varian 200 Mercury instrument (200 MHz) using $CDCl_3$ as a solvent and hexamethyldisiloxane (HMDSO) as internal standard. Mass spectra were registered on GC-MS HP 6890 (70 eV). GC analysis was performed on a Varian instrument equipped with flame-ionization detector using column packed with 5% OV-17 Chromosorb W-HP (80-100 mesh). Trifluroacetic anhydride was distillated prior use.

*ACYLATION OF 2-TRIMETHYLSILYLFURAN BY TRIFLUROACETIC ANHYDRIDE (method **a**).*

A mixture of 0.015 mol of 2-trimethylsilylfuran, 0.020 mol trifluoroacetic anhydride and 0.03 mmol $I_2$ was stirred for 32 h in a "Pierce" vial at 70°C. Process was controlled by GC until starting material disappeared. The mixture was cooled, supplemented with 10 ml of distilled water, stirred for 10 min and titrated with 10% KOH to pH 7. After washing with water solution of $Na_2S_2O_3$ and water, it was dried with anhydrous magnesium sulphate. The solvent was removed by distillation and the residue was fractionated in vacuum. A fraction boiling at 114-116°C/11mm Hg was collected giving 5-trimethylsilyl-2-trifluoroacetylfuran **(2)** (48%) as light yellow oil. $^1H$ NMR and mass spectral data of compound **2** are presented in Table 1.

*ACYLATION OF 2- TRIMETHYLGERMYLFURAN BY TRIFLUROACETIC ANHYDRIDE (method **b**).*

A mixture of 0.005 mol 2-trimethylgermylfuran and 0.0065 mol trifluoroacetic anhydride was stirred for 2 h in "Pierce" vial at 70°C. The excess of trifluoroacetic anhydride was removed under vacuum at room temperature. Aqueous saturated $NaHCO_3$ (4 ml) was added and the crude product was extracted with $Et_2O$ (3x15ml) and dried by $Na_2SO_4$. The solvent was removed and residue was purified by column chromatography using the mixture benzene-hexane (1:1) as eluent. Evaporation of eluent afforded the 5-

trimethylgermyl-2-trifluoroacetylfuran (4) (26%) as yellow oil. $^1$H NMR and mass spectral data of compound **4** are presented in Table 1.

Our attempts to synthesize the compound **4** by method a were unsuccessful. 5-*t*-Butyl-2-trifluoroacetylfuran (**1**) was prepared and isolated by method ***b*** (eluent hexane), the compounds **3** and **5** - by method ***a***.

**Pharmacology**

The neurotropic activity was studied on ICR-JCL mice (19-23 g). Ambient temperature (22 °C) was maintained in the laboratory and in the animal colony. The tested substances were administered intraperitoneally (*i.p.*) as aqueous suspensions prepared with the aid of Tween 80, 30 or 60 min prior to the assay. Control animals received injections of equal amounts of distilled water with Tween 80. Tests were performed according to Ref.13.

Conventional reflex of passive avoidance was applied to evaluate the influence of the substances in question on memory and antiamnesic activity. Retrogradal amnesia was caused transcorneally by maximal electric shock administered just after learning.

*IN VITRO* CYTOTOXICITY ASSAY. Monolayer cell lines were cultivated for 72 h in DMEM standard medium without an indicator and antibiotics. After the ampoule was defreezed not more than four passages were performed. The control cells and cells with tested substances in the range of 2-5 $10^4$ cell/mL concentration (depending on line nature) were placed on separate 96 wells plates. Solutions containing test compounds were diluted and added in wells to give the final concentrations of 50, 25, 12,5 and 6.25 μg/mL. Control cells were treated in the same manner only in the absence of test compounds. Plates were cultivated for 72 h. A quantity of survived cells was determined using crystal violet (CV) or 3-(4,5-dimethylthiazol-2-yl)-2,5-diphenyltetrazolinium bromide (MTT) coloration which was assayed by multiscan spectrophotometer. The quantity of alive cells on control plate was taken in calculations for 100% [14, 15]. Concentration of NO was determined according to [14].

### RESULTS AND DISCUSSION

Trifluoroacetylations of trialkyl(2-furyl)silanes, –germanes and their carbon analogue were realized under Friedel-Crafts conditions using trifluroacetic anhydride. Yields and reaction conditions are summarized in Table 1.

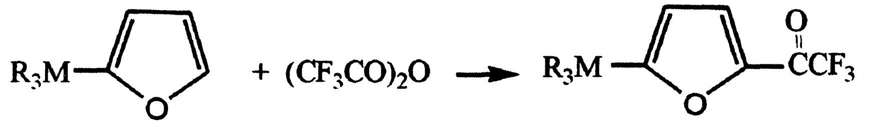

M = C, Si, Ge; R = Me, Et **1-5**

Table 1. Synthesis of Trifluoroacetylfurans **1-5**

| No. | $R_3M$ | Reaction time, h | T, °C | Yield, % | $^1$H NMR | MS-GC (m/z) |
|---|---|---|---|---|---|---|
| **1.** | $Me_3C$ | 24 | 20 | 72 | 1.33(s,9H,CMe$_3$), 6.29 (d,1H, H$^4$), 7.41(m,1H,H$^3$) | 220 (M$^+$,10), 205(M$^+$-Me,100) |
| **2.** | $Me_3Si$ | 32 | 70 | 48 | 0.35(s,9H,SiMe$_3$), 6.82 (d,1H, H$^4$), 7.50(m,1H, H$^3$) | 236 (M$^+$,30), 221(M$^+$-Me,90), 167(30),143(100) |
| **3.** | $Et_3Si$ | 26 | 70 | 51 | 0.44-1.27(m,15H,SiEt$_3$), 6.78(d,1H,H$^4$),7.56(m,1H,H$^3$) | 278(M$^+$,17), 249(M$^+$-Et,100), 221(90), 171(50) |
| **4.** | $Me_3Ge$ | 2 | 70 | 26 | 0.49(s,9H,GeMe$_3$), 6.74 (d,1H, H$^4$), 7.48(m,1H,H$^3$) | 282 M$^+$, 10), 267(M$^+$-Me,100) 123(50), 97(40) |
| **5.** | $Et_3Ge$ | 1 | 55 | 64 | 0.89-1.05(m,15H,GeEt$_3$), 6.76(d,1H,H$^4$),7.52(d,1H,H$^3$) | 324(M$^+$,5), 295(M$^+$-Et,100),267(50), 239(66),97(20) |

Biological investigations have demonstrated that the toxicity of trifluoroacetylfuran derivatives **1-5** strongly depends on the substituent at the position 5. Germanium derivatives **4,5** are more toxic compounds than corresponding silicon derivatives **2,3.** 5-Triethylgermyl-2-trifluoroacetylfuran (**5**) was the most toxic compound ($LD_{50}$ 11.2 mg $kg^{-1}$), 200 times more toxic than the silicon analogue (Table 2). It is interesting to note that trimethyl derivatives of silicon and their germanium analogues have comparable toxicity, but substitution of the methyl group by ethyl dramatically changes the toxicity (see Table 2). The neurotropic activity of 5-trialkylgermyl-2-trifluoroacetylfurans depends on the alkyl substituent at the germanium atom: the 5-triethylgermyl derivative **5** exibits the highest activity in the hexobarbital anaesthesia test and prolongs its duration by 137% (Table 2). The 2-trimethylgermyl derivative **4** exhibits a stimulating activity in the ethanol anaesthesia and completely prevents animals from retrogradal amnesia (RA) (Table 2).

Table 2. Acute Toxicity and Neurotropic Activity of 

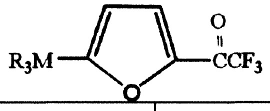

| $R_3M$ | $LD_{50}$, mg $kg^{-1}$ | Hypoxia % | Corazol induced spasms, % | Phenamine, stereotype, % | Anaesthesia, % of control | | RA, % |
|---|---|---|---|---|---|---|---|
| | | | | | Hexo-barbital | Ethanol | |
| $Me_3C$ (**1**) | 22 | 120 | 175 | 61 | 133 | 320 | 60 |
| $Me_3Si$ (**2**) | 112 | 100 | 224.7 | 44 | 86 | 240 | 16.7 |
| $Et_3Si$ (**3**) | 2240 | 128 | 94 | 90 | 89 | 74 | 50 |
| $Me_3Ge$ (**4**) | 71 | 113 | 149 | 58 | 96 | 51 | 100 |
| $Et_3Ge$ (**5**) | 11.2 | 109 | 129 | 84 | 237 | 71 | 80 |

Table 3. Effects of Trifluoroacetylfurans **1-5** on Locomotor Coordination and Muscle Tone

| $R_3M$ | $ED_{50}$ (mg $kg^{-1}$) | | | | |
|---|---|---|---|---|---|
| | Test: | | | | |
| | Rotating rod | Tube | Traction | Hypothermia | Analgesia |
| $Me_3C$ (**1**) | 4 (3-6) | 9 (6-12) | 9 (6-12) | 10 (7-14) | 10 (7-14) |
| $Me_3Si$ (**2**) | 4.1 (2.7-5.5) | 2.8 (1.8-3.7) | 35.5 (20.2-50.8) | 4.5 (3.1-6.0) | 28.2 (15.9-41.9) |
| $Et_3Si$ (**3**) | 447 (31.3-59.6) | 89 (63.1-119.6) | >250 | 112 (79-147) | >250 |
| $Me_3Ge$ (**4**) | 7 (50-93) | 14 (7-21) | 14 (7-21) | 7 (5-9) | 14 (5-26) |
| $Et_3Ge$ (**5**) | >5 | >5 | >5 | 11.5 (8-15) | 0.9 (0.6-1.2) |

The silicon and carbon analogues **1,2** prolonged the ethanol anaesthesia by 140 and 220%, correspondingly. They had higher anti-Corazol potency but they did not prevent animals from retrogradal amnesia. The pharmacological effects of phenamine are diminished by all the trifluroacetyl derivatives (Table 2). The effect of trifluoroacetylfuran derivatives **1-5** on locomotor coordination and muscle tonus depends on the substituent and differs to some extent (Table 3). Thus, compounds **1**, **2** nd **4** in rotating-rod, tube and traction tests have $ED_{50}$ in the 4 - 35.5 mg $kg^{-1}$ range, but compound **3** in the range 89 - 447 mg $kg^{-1}$. The 5-trimethylsilyl-2-fluoroacetylfuran (**2**) has the highest hypothermic action, but the 5-triethylgermyl derivative **5** has the highest analgesic activity ($EC_{50}$ 0.9 mg kg-1) (Table 3).

Table 4. Cytotoxicity of 

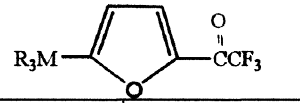

| Cell line | Method | $R_3M$ | | | | |
|---|---|---|---|---|---|---|
| | | $Me_3C$ (**1**) | $Me_3Si$ (**2**) | $Et_3Si$ (**3**) | $Me_3Ge$ (**4**) | $Et_3Ge$(**5**) |
| HT-1080 | CV | nce | nce | nce | nce | 72* |
| | MTT | nce | nce | nce | nce | 72 |
| | **NO | 4 | 6 | 5 | 18 | 250 |
| MG-22A | CV | nce | nce | nce | nce | 6 |
| | MTT | nce | nce | nce | nce | 10 |
| | NO | 5 | 7 | 15 | 14 | 250 |
| Neuro 2A | CV | nce | - | - | nce | 3.3 |
| | MTT | nce | | | nce | 4 |
| | NO | 5 | | | 18 | 300 |

* $IC_{50}$ (μg $mL^{-1}$) providing 50% cell killing effect (CV: coloration; MTT: coloration);
**NO – concentration (%) (CV: coloration);
nce - no cytotoxic effect; - Not tested.

Potential cytotoxic activity of synthesized compounds **1-5** was tested *in vitro* on three monolayer tumor cell lines: MG-22A (mouse hepatoma), HT-1080 (human fibrosarcoma), Neuro 2A (mouse neuroblastoma). Concentrations providing 50% of tumor death effect ($IC_{50}$) were determined according to the known procedure [16] using 96 well plates.

The compounds **1-5** have low activity on HT-1080, MG-22A and Neuro 2A tumor cell cultures (Table 4). The most active antitumor substance is 5-triethylgermyl-2-trifluoroacetylfuran ($IC_{50}$ on Neuro 2A 3.3 μg $ml^{-1}$). Germyl derivative **5** is stronger tumor growth inhibitor and NO-inducer than corresponding silyl analogue **2**.

**ACKNOWLEGMENT**
We are grateful to Latvian Taiho Foundation for financial support.

**REFERENCES**

1. N.A. Zaitseva, E.M. Panov, K.K. Kocheshkov, *Izv.Akad. Nauk USSR*, 1961, **5**, 831.
2. V.G. Glukhovtsev, J.V. Ilyin, A.V. Ignatenko, L.Yu. Breznev, *Izv.Akad. Nauk USSR, ser.khim.*, 1987, **12**, 2834.
3. W.S. DiMenna, P.M. Gross, *Tetrahedron Lett.*, 1980, 2129.
4. J.W. Guiles, *Synlett*, 1995, 165.
5. X. Creary, *J.Org.Chem.*, 1987, **52**, 5026.
6. F.A.J. Kerdesky, A.Basha, *Tetrahedron Let.*, 1991, 2003.
7. J.-P.Begue, D. Bonnet-Delpon, *Tetrahedron*, 1991, **47**, 3207.
8. C. Eaborn, *J. Organomet. Chem.*, 1975, **100**, 43.
9. W.P. Weber, *Silicon Reagents for Organic Synthesis*, Springer Verlag, Berlin-Heidelberg-New York, 1983.
10. *Drugs of the Future*,1994, **19**, 854.
11. J. Dow, B.D. Dulery, J.-M. Hornsperger, G.F. Di Francesco, P.Keshary, K.D. Haegele, *Arzneim.-Forsch/Drug.Res.* 1995, 45(II), Nr.12, 1245.
12. X. Zhu, E. Giacobini, J.-M. Hornsperger, *Eur. J. Pharmacol.*, 1995, **275**, 93.
13. Lukevics, L. Ignatovich, N. Porsiurova, S. Germane, *Appl. Organomet. Chem.*, 1988, **2**, 115.
14. D.J. Fast, R.C. Lynch, R.W. Leu, *J. Leuckocyt. Biol.*, 1992, **52**, 255.
15. P.J. Freshney, *Culture of Animal Cells (A Manual of Basic Technique)*, Wiley-Liss, New York, 1994, pp.296-297.
16. R.J. Riddell, R.H. Clothier, M.F. Balls, *Chem. Toxicol.*, 1986, **24**, 469.

